# Coronary artery status of patients with transient fever 24–36 h after first IVIG infusion did not differ from that seen in responsive patients

**DOI:** 10.1186/s12969-018-0301-6

**Published:** 2018-12-29

**Authors:** Jae Suk Baek, Jeong Jin Yu, Mi Jin Kim, Jihye You, Hyun Ok Jun, Young-Hwue Kim, Jae-Kon Ko

**Affiliations:** 1 Department of Pediatrics, University of Ulsan College of Medicine, Seoul, South Korea; 2 Division of Pediatric Cardiology, Department of Pediatrics, Asan Medical Center, 88 Olympic-ro 43-gil, Songpa-gu, Seoul, 05505 South Korea

**Keywords:** Kawasaki disease, Intravenous immunoglobulin, Unresponsive, Fever, Prediction

## Abstract

**Background:**

Current management guidelines for patients with Kawasaki disease (KD) differ in their recommendations for fever observation times when determining resistance to initial intravenous immunoglobulin (IVIG). This retrospective study assessed coronary artery status in patients with transient fever 24–36 h after the completion of a first IVIG infusion.

**Methods:**

Children with KD treated with IVIG between January 2006 and February 2017 were included. Subjects were divided into three groups according to response following the completion of initial IVIG treatment (Group 1, no fever after 24 h; Group 2, transient fever at 24–36 h; Group 3, others).

**Results:**

A total of 879 children were evaluated (Group 1, *n* = 663; Group 2, *n* = 54; Group 3, *n* = 162). During the subacute phase, the left main coronary artery (LMCA) diameter z score in both groups was significantly lower than that in Group 3 (Group 1: 1.02, Group 2: 0.87, Group 3: 1.24; Group 1 vs 2, *P* = 0.298; Group 1 vs 3, *P* = 0.025; Group 2 vs 3, *P* = 0.042); similar results were seen with the left anterior descending coronary artery (LAD) diameter z score (Group 1: 0.64, Group 2: 0.38, Group 3: 0.98; Group 1 vs 2, *P* = 0.083; Group 1 vs 3, *P* = 0.001; Group 2 vs 3, *P* = 0.004). The coronary artery (CA) status also did not differ between Groups 1 and 2 during the convalescent phase (z score of LMCA was 0.70 in Group 1 and 0.74 in Group 2, *P* = 0.790; z score of LAD was 0.42 and 0.46 respectively, *P* = 0.796; z score of right CA was 0.07 and 0.00 respectively, *P* = 0.630). A multivariate logistic regression analysis identified total bilirubin level (OR, 2.472; 95% CI, 1.284–4.762; *P* = 0.007) as the only significant predictor of persisting fever over 36 h in patients with fever 24 h after the completion of initial IVIG.

**Conclusions:**

The CA status of patients with transient fever 24–36 h after the first IVIG infusion did not differ from that seen in responsive patients.

## Introduction

Kawasaki disease (KD) is an acute febrile illness of unknown cause that predominantly affects children under the age of 5 years [1]. In developed countries, KD is currently the most commonly acquired heart disease in children [1]. Although timely administration of intravenous immunoglobulin (IVIG) has reduced the incidence of coronary artery aneurysm (CAA) from 25 to 4% [1], approximately 10–20% of patients with KD are resistant to initial IVIG treatment [2, 3]. Resistant patients are, therefore, reported to be at increased risk of developing CAA [4–6]. However, the definition of resistance (based on resolution of fever) differs between two major management guidelines; the Japanese Society of Pediatric Cardiology and Cardiac Surgery (JCS) suggest an observation period of 24 h after completion of initial IVIG, whereas the American Heart Association (AHA) propose an observation period of 36 h [1, 7].

The purpose of this study was to investigate coronary artery parameters in patients with transient fever 24–36 h after the administration of initial IVIG by evaluating coronary artery status. A predictor of persisting fever over 36 h was additionally investigated.

## Materials and methods

### Subjects

The study included consecutive children admitted to the Asan Medical Center for the treatment of acute KD between January 2006 and February 2017. Many of subjects lived in the southeastern districts of Seoul, Korea. Patients were excluded if their fever spontaneously subsided before initial IVIG was administered, if they had been transferred to the Asan Medical Center from another center (therefore lacking a detailed record of early clinical features), and if the echocardiographic data of coronary artery status were incomplete.

This study was approved by the Institutional Review Board of the Asan Medical Center (2018–0004); the requirement for informed patient consent was waived due to the retrospective study design.

### Data acquisition

The medical records of subjects were retrospectively reviewed to obtain demographic, clinical, and laboratory data, as well as coronary artery diameter measurements. A diagnosis of KD was made in accordance with the AHA guidelines [1].

Fever was defined as an axillary body temperature ≥ 37.5 °C. Subjects were grouped according to the presence and timing of fever: Group 1, patients without fever 24 h after IVIG treatment; Group 2, those with transient fever 24–36 h after treatment; Group 3, the others with persistent fever over 36 h or recrudescent fever 36 h after treatment.

The plasma level of brain natriuretic peptide (BNP) was log transformed (log-BNP) for statistical analysis. A diagnosis of pyuria was made by microscopic examination of urine samples (> 10 white blood cells per high power field).

All echocardiographic examination was performed by one author (JJ Yu). Coronary artery diameter was measured during the subacute (between 2 weeks and 1 month after the onset of illness) and convalescent (at 2 months after the onset of illness) phases of KD; the highest value of measurement of diameter was recorded and converted to a z score as previously reported [8]. A z score ≥ 2.5 for any coronary artery was determined to indicate the presence of CAA [1, 9]. In addition, we have once again defined CAA according to the criteria of the Japanese Ministry of Health and Welfare as a coronary artery diameter ≥ 3 mm in children < 5 years old and ≥ 4 mm in children ≥5 years old [10].

### Statistical analysis

All data are presented as frequency (%) or mean ± standard deviation. The Chi-square or Student’s T test was used as appropriate. Univariate logistic regression analysis for the prediction of persistent fever was performed using data from patients in Group 2 and patients in Group 3 with fever starting between 24 and 36 h after completion of the first IVIG infusion. Multivariate analysis was performed using variables identified as statistically significant in the univariate analysis. In addition, receiver operating characteristic curve (ROC) analysis was performed for any identified predictors. SPSS version 21.0 (IBM Co., Armonk, NY, USA) was used for all statistical analysis. Statistical significance was defined as a *P* value < 0.05.

## Results

### Subjects

**Fig. 1:**
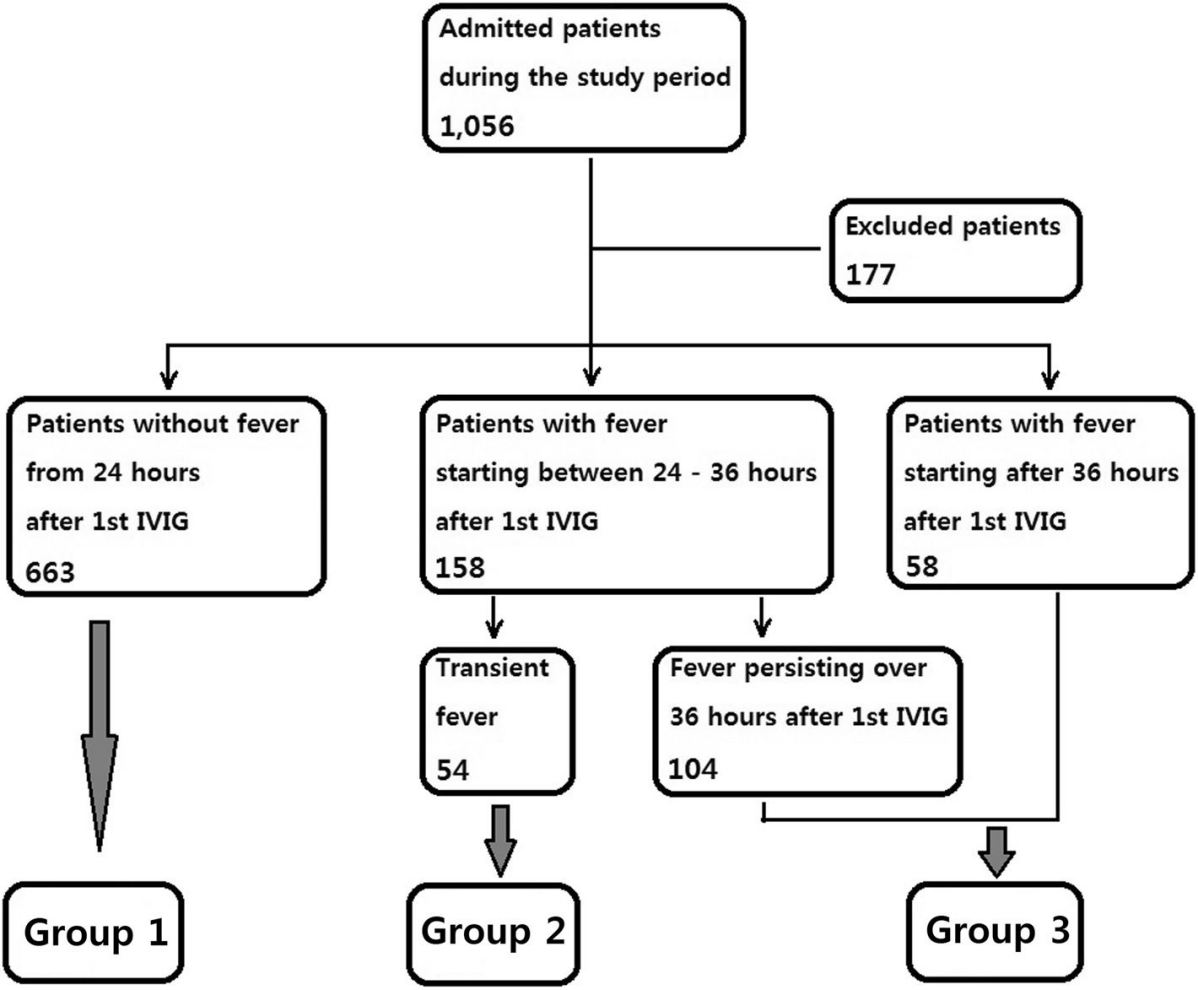
Patient enrollment and classification

Patient enrollment and classification is summarized in Fig. 1. A total of 1056 patients were admitted during the study period; 177 were excluded from the analysis (102 due to spontaneous resolution of fever prior to IVIG administration, 11 due to incomplete records of early clinical features, and 61 due to incomplete data on coronary artery status). Therefore, 879 patients were included in the analysis, 663 (75.4%) in Group 1, 54 (6.1%) in Group 2, and 162 (18.4%) in Group 3. In Group 3, fever persisted from 24 h to beyond 36 h in 104 patients and started from 36 h in 58. All subjects received a 2 g/kg dose of IVIG as the initial treatment during the acute phase of illness.

### Comparison of clinical and coronary characteristics between groups

The mean age of subjects in Group 1 was 2.43 years, which was lower than that seen in Group 3 (2.85 years, *P* = 0.014; Table 1). Body weight and height were also lower in Group 1 than Group 3 (*P* = 0.009 and *P* = 0.016, respectively). There were no significant differences between Groups 2 and 3 in age (*P* = 0.839), body weight (*P* = 0.663), or height (*P* = 0.784). Male gender was more frequent in Group 3 than in Group 1 (66.0% vs 57.0%, respectively; *P* = 0.041). Although the proportion of male patients appeared to be lower in Group 2 (53.7%) than Group 3 (66.0%), this difference did not reach statistical significance (*P* = 0.108). No statistically significant differences were seen between the three groups in terms of clinical presentation, frequency of BCG site reactions, and treatment duration.

**Table 1:** Patient characteristics

Characteristics	Group 1 *n* = 663	Group 2 *n* = 54	Group 3 *n* = 162	*P* value		
				1 vs 2	1 vs 3	2 vs 3
Age, years	2.43 ± 1.88	2.91 ± 1.91	2.85 ± 2.07	0.072	0.014	0.839
Male	378 (57.0)	29 (53.7)	107 (66.0)	0.670	0.041	0.108
Body weight, kg	12.9 ± 4.9	14.4 ± 5.1	14.1 ± 5.2	0.032	0.009	0.663
Height, cm	88.8 ± 16.7	93.0 ± 15.4	92.4 ± 16.2	0.075	0.016	0.784
*Diagnostic criteria*						
Conjunctivitis	640 (96.5)	50 (92.6)	154 (95.1)	0.138	0.361	0.500
Red lips/oral mucosa	606 (91.4)	47 (87.0)	145 (89.5)	0.315	0.445	0.621
Rash	566 (85.4)	50 (92.6)	147 (90.7)	0.159	0.075	0.788
Cervical lymphadenopathy	406 (61.2)	38 (70.4)	111 (68.5)	0.194	0.103	0.866
Changes in extremities	563 (84.9)	47 (87.0)	144 (88.9)	0.843	0.213	0.806
Complete presentation	565 (85.2)	48 (88.9)	144 (88.9)	0.551	0.258	1.000
BCG site reaction	313 (47.2)	21 (38.9)	66 (40.7)	0.259	0.159	0.873
Treatment duration, days	6.0 ± 1.2	5.8 ± 1.3	5.9 ± 1.2	0.174	0.441	0.424
*Laboratory findings*						
WBC, ×10^3^/μL	14.6 ± 4.9	14.4 ± 4.8	14.3 ± 5.4	0.855	0.600	0.901
Neutrophil, %	63.3 ± 13.9	66.6 ± 15.2	70.9 ± 14.0	0.094	< 0.001	0.061
Hemoglobin, g/dL	11.3 ± 1.1	11.2 ± 1.0	11.2 ± 1.1	0.828	0.631	0.940
Platelet, ×10^3^/μL	338.0 ± 99.9	316.6 ± 89.4	322.8 ± 107.1	0.128	0.088	0.702
Protein, g/dL	6.6 ± 0.7	6.7 ± 0.5	6.6 ± 0.8	0.835	0.795	0.732
Albumin, g/dL	3.4 ± 0.5	3.4 ± 0.3	3.2 ± 0.5	0.636	< 0.001	0.010
AST, IU/L	81.5 ± 140.6	140.6 ± 249.3	144.9 ± 284.7	0.006	< 0.001	0.922
ALT, IU/L	93.5 ± 139.0	134.3 ± 209.1	150.59 ± 200.6	0.048	< 0.001	0.611
Total bilirubin, mg/dL	0.72 ± 0.74	0.65 ± 0.50	1.21 ± 1.23	0.496	< 0.001	0.001
Na^+^, mmol/L	136.1 ± 2.6	136.3 ± 2.6	135.3 ± 3.2	0.575	0.001	0.041
C-reactive protein, mg/dL	8.63 ± 6.18	8.74 ± 7.16	10.91 ± 7.10	0.898	< 0.001	0.054
BNP, pg/mL	108.0 ± 221.2	107.8 ± 286.3	143.9 ± 223.3			
Log-BNP	1.65 ± 0.56	1.55 ± 0.59	1.77 ± 0.58	0.264	0.023	0.027
Pyuria	189 (28.5)	16 (29.6)	57 (35.2)	0.875	0.150	0.615
*Coronary artery diameter during subacute phase*						
LMCA, mm	2.33 ± 0.44	2.36 ± 0.40	2.48 ± 0.52			
Z score	1.02 ± 1.08	0.87 ± 1.02	1.24 ± 1.22	0.298	0.025	0.042
LAD, mm	1.85 ± 0.40	1.83 ± 0.31	2.05 ± 0.67			
Z score	0.64 ± 1.07	0.38 ± 0.90	0.98 ± 1.40	0.083	0.001	0.004
RCA, mm	1.83 ± 0.48	1.92 ± 0.34	2.03 ± 0.68			
Z score	0.37 ± 1.29	0.44 ± 0.85	0.73 ± 1.59	0.710	0.003	0.197
CAA on z score (subacute)	81 (12.2)	5 (9.3)	27 (16.7)	0.665	0.152	0.268
CAA on Japanese criteria (subacute)	37 (5.6)	1 (1.9)	11 (6.8)	0.350	0.574	0.302
*Coronary artery diameter during conv. Phase*						
LMCA, mm	2.24 ± 0.40	2.33 ± 0.38	2.35 ± 0.40			
Z score	0.70 ± 1.10	0.74 ± 1.10	0.84 ± 1.07	0.790	0.139	0.552
LAD, mm	1.79 ± 0.33	1.86 ± 0.24	1.92 ± 0.53			
Z score	0.42 ± 1.02	0.46 ± 0.67	0.59 ± 1.14	0.796	0.076	0.440
RCA, mm	1.76 ± 0.36	1.80 ± 0.32	1.92 ± 0.65			
Z score	0.07 ± 1.07	0.00 ± 0.89	0.32 ± 1.50	0.630	0.014	0.135
CAA on z score (conv.)	38 (5.7)	5 (9.3)	20 (12.3)	0.363	0.006	0.681
CAA on Japanese criteria (conv.)	16 (2.4)	1 (1.9)	7 (4.3)	1.000	0.187	0.683

Data are reported as mean ± standard deviation, or number (%)

*WBC* white blood cell, *ESR* erythrocyte sedimentation rate, *AST* aspartate aminotransferase, *ALT* alanine aminotransferase, *BNP* brain natriuretic peptide, *LMCA* left main coronary artery, *LAD* left anterior descending, *RCA* right coronary artery, *CAA* coronary artery aneurysm, *conv*. convalescent

Comparison of laboratory data obtained prior to first IVIG infusion showed the neutrophil percentage and serum C-reactive protein levels to be higher in Group 3 than in Group 1 (*P* <  0.001 and *P* <  0.001, respectively). Serum albumin levels were lower in Group 3 (Group 1 vs 3, *P* < 0.001; Group 2 vs 3, *P* = 0.010), as were sodium concentrations (Group 1 vs 3, *P* = 0.001; Group 2 vs 3, *P* = 0.041).

Serum total bilirubin levels were higher in Group 3 than in Groups 1 and 2 (Group 1 vs 3, *P* < 0.001; Group 2 vs 3, P = 0.001), as were log-BNP levels (Group 1 vs 3, *P* = 0.023; Group 2 vs 3, *P* = 0.027). Liver enzyme levels were higher in Groups 2 and 3 than Group 1 (aspartate aminotransferase: Group 1 vs 2, *P* = 0.006; Group 2 vs 3, P < 0.001; alanine aminotransferase: Group 1 vs 2, *P* = 0.048; Group 2 vs 3, *P* < 0.001).

**Fig. 2:**
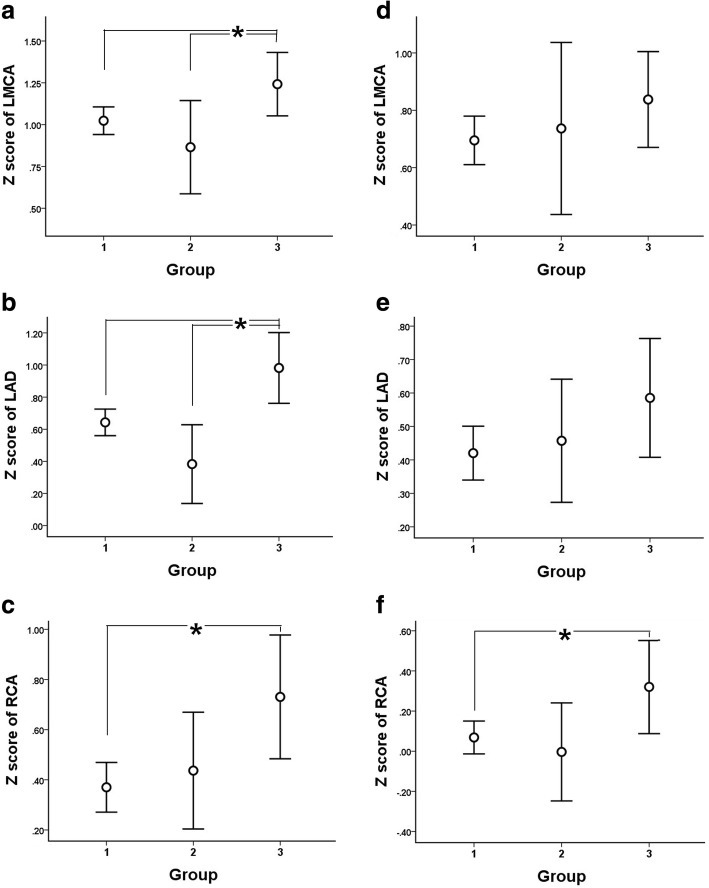
Z score of coronary arteries in the three groups during the subacute (**a**–**c**) and convalescent phases (**d**–**f**). LMCA, left main coronary artery; LAD, left anterior descending coronary artery; RCA, right coronary artery. *P value < 0.05

**Fig. 3:**
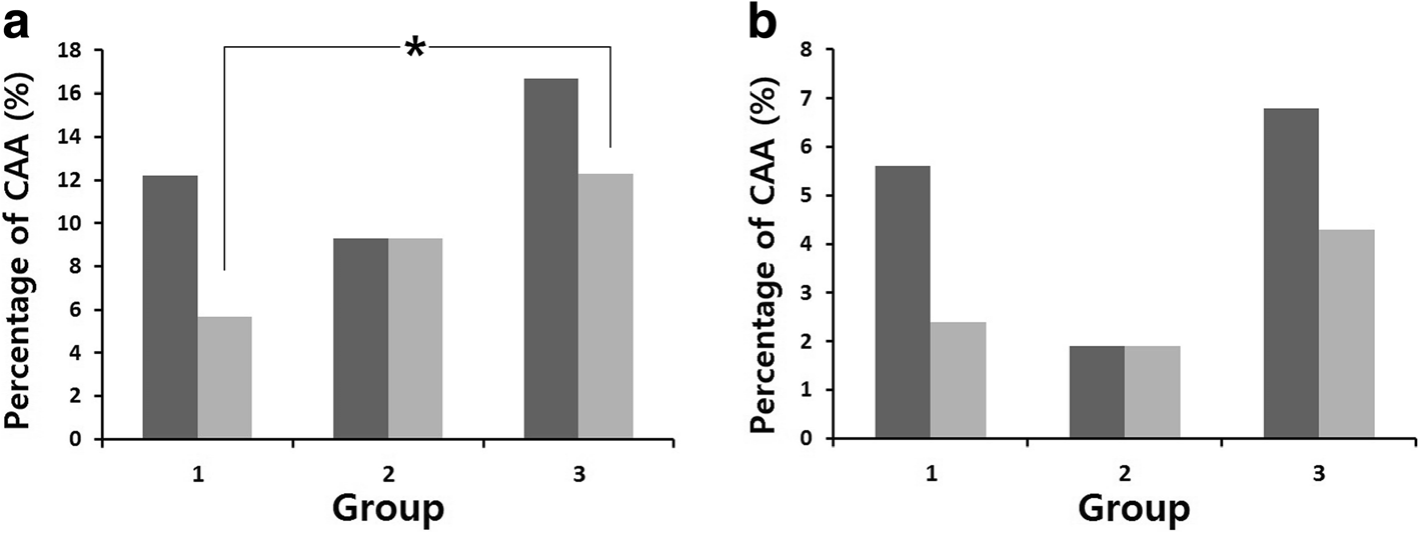
Percentage of subjects with CAA in the three groups, based on **a**) z score and **b**) Japanese criteria. Dark gray color for subacute phase and light gray color for convalescent phase. *P value < 0.05

A comparison of coronary artery diameter and CAA frequency z scores is shown in Table 1 and Figs. 2 and 3. No significant differences were seen between Groups 1 and 2. During the subacute phase, the mean left main coronary artery (LMCA) diameter was larger in Group 3 than in Groups 1 and 2 (Group 3 vs 1, P = 0.023; Group 3 vs 2, *P* = 0.042). Similar results were seen with the left anterior descending (LAD) coronary artery diameter (Group 3 vs 1, *P* = 0.001; Group 3 vs 2, *P* = 0.004). The mean diameter of the right coronary artery (RCA) was larger in Group 3 than Group 1 (*P* = 0.003). There were, however, no significant differences in the frequency of CAA between the three groups.

During the convalescent phase, the diameter of the RCA and the frequency of CAA based on z scores were higher in Group 3 than in Group 1 (*P* = 0.014 and *P* = 0.006, respectively).

### Logistic regression analyses for the prediction of persisting fever

**Fig. 4:**
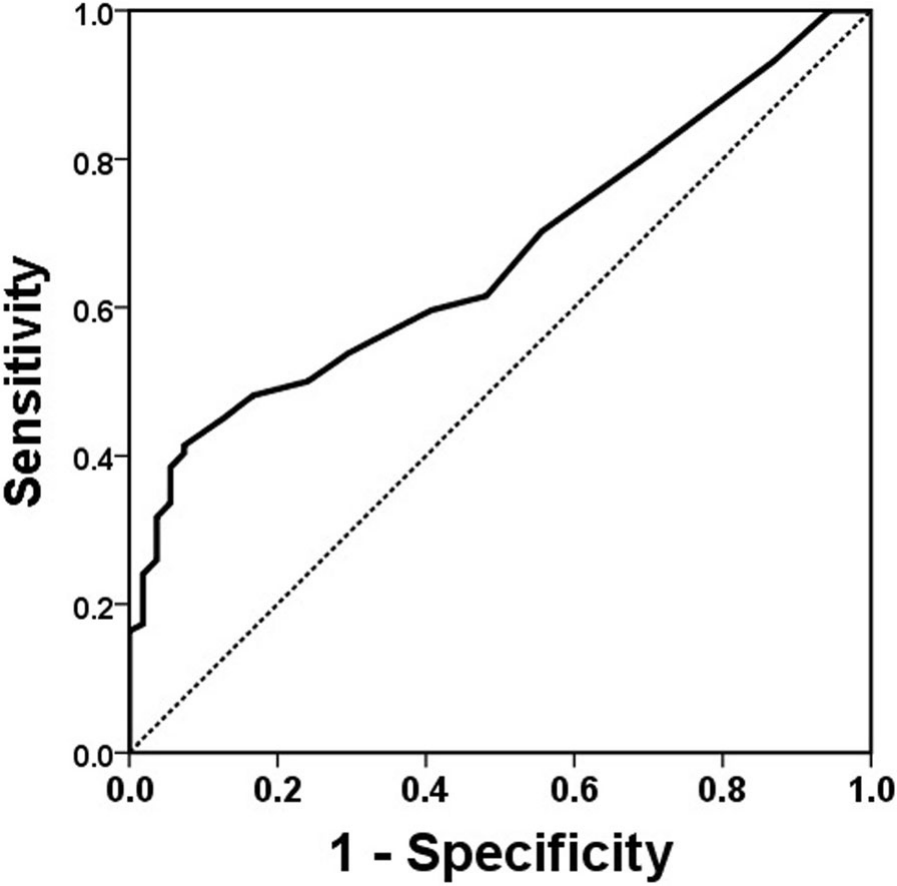
Receiver operating characteristic curve analysis of the serum bilirubin level as a predictor of fever persisting beyond 36 h

Data from Group 2 plus the Group 3 patients with fever starting at 24–36 h and persisting beyond 36 h were included in the logistic regression analysis (*n* = 158). As shown in Table 2, univariate analysis identified significant predictors of persistent fever beyond 36 h after completion of IVIG therapy to be neutrophil percentage (OR, 1.027; 95% CI, 1.003–1.052; *P* = 0.025), serum albumin level (OR, 0.409; 95% CI, 0.195–0.858; *P* = 0.018), total bilirubin level (OR, 2.621; 95% CI, 1.537–4.469; *P* < 0.001), sodium concentration (OR, 0.848; 95% CI, 0.753–0.955; *P* = 0.007), C-reactive protein level (OR, 1.063; 95% CI, 1.009–1.119; *P* = 0.022), and log-BNP level (OR, 2.220; 95% CI, 1.132–4.354; *P* = 0.020). However, multivariate analysis identified total bilirubin level (OR, 2.472; 95% CI, 1.284–4.762; *P* = 0.007) as the only significant predictor. The ROC analysis for serum bilirubin level is shown in Fig. 4. The area under the curve was 0.668 (95% CI, 0.585–0.751); the sensitivity was 41.3% and the specificity was 92.6% at the cutoff level of total bilirubin level > 1.15 mg/dL.

**Table 2:** Logistic regression analysis for the prediction of persistent fever in 158 patients with fever starting 24–36 h after the completion of initial IVIG therapy

Characteristics	Group 2 *n* = 54	Group 3 *n* = 104	Univariate			Multivariate		
			*P* value	OR	CI (95%)	*P* value	OR	CI (95%)
Neutrophil, %	66.6 ± 15.2	72.1 ± 13.4	0.025	1.027	1.003–1.052	0.951	1.001	0.970–1.033
Albumin, g/dL	3.4 ± 0.3	3.2 ± 0.5	0.018	0.409	0.195–0.858	0.109	0.422	0.147–1.212
Total bilirubin, mg/dL	0.65 ± 0.50	1.43 ± 1.33	< 0.001	2.621	1.537–4.469	0.007	2.472	1.284–4.762
Na^+^, mmol/L	136.3 ± 2.6	134.9 ± 3.1	0.007	0.848	0.753–0.955	0.803	1.022	0.863–1.209
C-reactive protein, mg/dL	8.74 ± 7.16	11.66 ± 7.40	0.022	1.063	1.009–1.119	0.884	0.995	0.935–1.060
Log-BNP	1.55 ± 0.59	1.81 ± 0.59	0.020	2.220	1.132–4.354	0.493	1.316	0.600–2.887

Data are reported as mean ± standard deviation or number (%)

*OR* Odds ratio, *CI* confidence interval, *BNP* brain natriuretic peptide

## Discussion

Key guidelines differ in their recommendations for the definition of resistance to initial IVIG therapy, with the JCS recommending a 24 h observation period and the AHA suggesting a 36 h observation period from the end of treatment [1, 7]. In this study, patients with transient fever 24–36 h after the completion of first IVIG (Group 2) were compared with the other patient groups in terms of clinical characteristics and coronary artery status. The characteristics of subjects in Group 2 appeared to be more similar to those of subjects in Group 1 than Group 3, despite differences in body weight and liver transaminases between the two groups. Regarding other laboratory variables that reflect the degree of inflammation during the acute phase (white blood cell count, neutrophil percentage, C-reactive protein level, and hemoglobin level) [11], the values in Group 2 did not differ from those in Group 1. Subjects in Groups 1 and 2 showed higher levels of serum albumin and sodium and lower levels of total bilirubin and log-BNP than subjects in Group 3.

Univariate analysis showed that the neutrophil percentage, serum albumin level, total bilirubin level, sodium level, C-reactive protein level, and log-BNP were significant predictors of fever persisting beyond 36 h after completion of the first IVIG infusion. This is consistent with the results of previous studies of predictors of IVIG resistance [12–15]. However, multivariate analysis identified only a single predictor - total bilirubin level which has been reported to be a predictor of resistance to initial IVIG treatment [14, 16]. In this study, it was the only one predictor of persisting fever beyond 36 h in subjects with fever 24 h after initial treatement. However, its use in clinical practice will be limited due to the low sensitivity (41.3%).Analysis of coronary artery status showed no differences between Groups 1 and 2. During the subacute phase, the z scores of the diameter of LMCA and LAD in both Group 1 and Group 2 were significantly lower than those in Group 3. The coronary artery (CA) status also did not differ between Groups 1 and 2 during the convalescent phase. Based on these results, a 36 h period of observation after IVIG can be considered to be reasonable. With the JCS guidelines, the patients in group 2 would have received an unnecessary additional IVIG treatment. IVIG is a biological product made from pooled donor plasma, and several adverse effects including hemolytic anemia were reported [1, 17]. Therefore, efforts should be made to avoid unnecessary IVIG administration.

In the group 2, the frequency of CAA looks not to be reduced in the convalescent phase. We cannot clearly explain this result but assume that it may be a coincident result by the relatively small number of subjects in group 2.

This study has several limitations. The first one is the retrospective nature of this study. The outcome of patients cannot be compared between different observation times because we consistently observed patients for 36 h during the study period. A prospective study involving randomly selected patients managed according to two different guidelines (24 vs 36 h) is recommended to determine the appropriate observation time after the first IVIG treatment. Alternatively, a study involving multiple institutes using different guidelines could be conducted. Second, the number of patients with transient fever 24–36 h after completion of the first IVIG treatment was small and the follow-up period continued only until the convalescent phase. Third, the possible benefit of an earlier second treatment after 24 h of observation, rather than after 36 h, could not be investigated in this study. One hundred four (65.8%) of the 158 patients with fever starting between 24 and 36 h after IVIG had fever persisting beyond 36 h. Finally, all subjects in this study were Korean, so the results need to be reproduced in other races.

## Conclusion

The outcome of patients with transient fever between 24 and 36 h after the completion of first IVIG treatment did not differ from that of responsive patients. A future prospective or multicenter study to determine the appropriate observation time after initial IVIG therapy is recommended based on the outcome of this study.

## Abbreviations

AHA: American Heart Association
BNP: Brain natriuretic peptide
CAA: coronary artery aneurysm
CI: Confidence Interval
IVIG: Intravenous immunoglobulin
JCS: Japanese Society of Pediatric Cardiology and Cardiac Surgery
KD: Kawasaki disease
LAD: Left anterior descending
LMCA: Left main coronary artery
log-BNP: log transformed BNP
RCA: Right coronary artery
ROC: Receiver operating characteristic curve

## References

[1] McCrindle BW, Rowley AH, Newburger JW, Burns JC, Bolger AF, Gewitz M (2017). Diagnosis, treatment, and long-term Management of Kawasaki Disease: a scientific statement for health professionals from the American Heart Association. Circulation.

[2] Nakamura Y, Yashiro M, Uehara R, Oki I, Watanabe M, Yanagawa H (2008). Epidemiologic features of Kawasaki disease in Japan: results from the nationwide survey in 2005-2006. Journal of epidemiology.

[3] Kim GB, Park S, Eun LY, Han JW, Lee SY, Yoon KL (2017). Epidemiology and clinical features of Kawasaki disease in South Korea, 2012-2014. Pediatr Infect Dis J.

[4] Burns JC, Capparelli EV, Brown JA, Newburger JW, Glode MP (1998). Intravenous gamma-globulin treatment and retreatment in Kawasaki disease. US/Canadian Kawasaki syndrome study group. Pediatr Infect Dis J.

[5] Durongpisitkul K, Soongswang J, Laohaprasitiporn D, Nana A, Prachuabmoh C, Kangkagate C (2003). Immunoglobulin failure and retreatment in Kawasaki disease. Pediatr Cardiol.

[6] Uehara R, Belay ED, Maddox RA, Holman RC, Nakamura Y, Yashiro M (2008). Analysis of potential risk factors associated with nonresponse to initial intravenous immunoglobulin treatment among Kawasaki disease patients in Japan. Pediatr Infect Dis J.

[7] Research Committee of the Japanese Society of Pediatric Cardiology; Cardiac Surgery Committee for Development of Guidelines for Medical Treatment of Acute Kawasaki Disease (2014). Guidelines for medical treatment of acute Kawasaki disease: report of the Research Committee of the Japanese Society of Pediatric Cardiology and Cardiac Surgery (2012 revised version). Pediatrics international : official journal of the Japan Pediatric Society.

[8] McCrindle BW, Li JS, Minich LL, Colan SD, Atz AM, Takahashi M (2007). Coronary artery involvement in children with Kawasaki disease: risk factors from analysis of serial normalized measurements. Circulation.

[9] Manlhiot C, Millar K, Golding F, McCrindle BW (2010). Improved classification of coronary artery abnormalities based only on coronary artery z-scores after Kawasaki disease. Pediatr Cardiol.

[10] Research Committee on Kawasaki Disease. Report of Subcommittee on Standardization of Diagnostic Criteria and Reporting of Coronary Artery Lesions in Kawasaki Disease (1984). Tokyo: Ministry of Health and Welfare.

[11] Tremoulet AH, Jain S, Chandrasekar D, Sun X, Sato Y, Burns JC (2011). Evolution of laboratory values in patients with Kawasaki disease. Pediatr Infect Dis J.

[12] Kobayashi T, Inoue Y, Takeuchi K, Okada Y, Tamura K, Tomomasa T (2006). Prediction of intravenous immunoglobulin unresponsiveness in patients with Kawasaki disease. Circulation.

[13] Egami K, Muta H, Ishii M, Suda K, Sugahara Y, Iemura M (2006). Prediction of resistance to intravenous immunoglobulin treatment in patients with Kawasaki disease. J Pediatr.

[14] Sano T, Kurotobi S, Matsuzaki K, Yamamoto T, Maki I, Miki K (2007). Prediction of non-responsiveness to standard high-dose gamma-globulin therapy in patients with acute Kawasaki disease before starting initial treatment. Eur J Pediatr.

[15] Sleeper LA, Minich LL, McCrindle BM, Li JS, Mason W, Colan SD (2011). Evaluation of Kawasaki disease risk-scoring systems for intravenous immunoglobulin resistance. The Journal of pediatrics.

[16] Fukunishi M, Kikkawa M, Hamana K, Onodera T, Matsuzaki K, Matsumoto Y (2000). Prediction of non-responsiveness to intravenous high-dose gamma γ-globulin therapy in patients with Kawasaki disease at onset. J Pediatr.

[17] Hamada H, Suzuki H, Abe J, Suzuki Y, Suenaga T, Takeuchi T (2012). Inflammatory cytokine profiles during cyclosporine treatment for immunoglobulin-resistant Kawasaki disease. Cytokine.

